# Fall-from-Bed Risk Prediction Using Physics-Based Bed Simulation

**DOI:** 10.3390/s26102979

**Published:** 2026-05-09

**Authors:** Jaeyong Kim, Hyeonwoo Kim, Jihwan Won, Jiwoon Lee, Hyeonjung Kim, Sunwoo Yeon, Ryanghee Sohn, Youngho Cho, Cheolsoo Park

**Affiliations:** 1 Department of Computer Engineering, Kwangwoon University, Seoul 01897, Republic of Korea; evejaeyong@kw.ac.kr (J.K.); woo@kw.ac.kr (H.K.); wjh5597486@kw.ac.kr (J.W.); jiwoonlee@kw.ac.kr (J.L.); a9016038@kw.ac.kr (H.K.); davidyeon123@kw.ac.kr (S.Y.); 2 EMMA Healthcare Corporation, Seongnam 13309, Republic of Korea; 3 Department of Information and Communication Engineering, Soonchunhyang University, Asan 31538, Republic of Korea

**Keywords:** health care, simulation, machine learning, regression, fall-from-bed, risk prediction

## Abstract

Fall-from-bed is a critical safety issue in hospitals and long-term care; however, large-scale real-fall data are rare, and collecting the data is ethically constrained. This study examined whether the fall-from-bed risk can be inferred from single static in-bed postures without temporal motion. We developed a physics-based bed–human simulator in MuJoCo and generated labeled episodes by sampling diverse initial configurations, rolling out uncontrolled dynamics for three seconds, and detecting falls by floor contact. Each initial state was represented as a 13-keypoint 2D skeleton in a bed-centric coordinate frame, normalized to fixed bed bounds, and supervised with a continuous risk label derived from time-to-fall using per-frame discounting on a 30 frame-per-second grid. On a pose-balanced simulated test set of 50,000 initial postures, the best-performing multilayer-perceptron-based predictor attained an area under the receiver operating characteristic curve of 0.9755, area under the precision–recall curve of 0.9771, F1-score of 0.9138, and mean squared error of 0.0374 (mean over five random seeds). Pose-stratified initialization improved performance relative to fully random sampling. Consistently high performance was observed across supine/prone/lateral subgroups, which improved with training set size. These results suggest that a static posture contains predictive information about fall risk under matched simulator dynamics, supporting the feasibility of posture-based risk scoring in the controlled settings.

## 1. Introduction

In elderly care and clinical environments, the bed serves as a primary living space, but it is also a potential source of safety risk [1,2,3]. Fall-from-bed incidents that occur when a patient changes posture or unintentionally moves to the bed edge can result in serious injuries, making them a critical safety concern in hospitals and long-term care facilities [1,4,5]. Consequently, sensor-based and artificial intelligence-based approaches to monitoring in-bed human states and the early assessment of fall risk have received increasing attention [6,7,8,9,10].

Studies related to bed environments focus on detecting falls after their occurrence or recognizing behaviors associated with bed exit [6,8,11]. For example, vision-based or depth-based sensing approaches have been proposed to detect fall-from-bed events [12], while other studies have used pressure sensors and visual information to recognize or infer bed-exit intentions [13,14,15]. These studies provide an important foundation for designing fall detection and prevention systems in real-world settings. However, the underlying problems are typically formulated around temporal motion sequences or event-based detection, requiring continuous observation of dynamic behaviors [15,16,17].

In contrast, analyzing fall risk from initial static configurations shifts the focus from event detection to risk estimation before motion. However, acquiring sufficient real-world data for fall-related analysis is challenging [18,19,20,21]. Fall-from-bed events are inherently difficult to capture using real-world data alone [22,23]. Falls are relatively rare, and intentionally inducing hazardous situations raises ethical and safety concerns [19,24]. For this reason, physics-based simulation has emerged as a useful alternative for generating data for bed-related fall analysis [25,26]. Simulation environments allow explicit modeling of bed structures and human bodies, enabling the safe generation of diverse initial postures and systematic outcome analysis.

Within simulation-based approaches, defining the initial human posture constitutes a key design choice [27,28]. Rather than assuming specific fall motions or predefined scenarios, this study generates initial human on-the-bed postures through random initial-state sampling, a commonly adopted strategy in physics-based simulation. This approach is relatively simple to implement and enables broad exploration of possible initial postures without imposing strong assumptions about particular movements or trajectories [29,30].

This study addresses the following problems: given a single posture of a human on a bed, can one predict whether it will eventually lead to a fall-from-bed event within three seconds? To investigate this question, we generate only the initial posture and then apply physics-based simulation without additional control or motion inputs, labeling each sample based on whether a fall from the bed occurs. These samples constitute a dataset that is used to formulate and analyze the fall risk regression problem that relies solely on the initial posture information. This formulation differs from that of existing studies in that it focuses on initial-state-based fall risk assessment rather than post-fall motion recognition or sequence-based analysis [31,32,33]. The model is trained as a supervised regression model using a continuous target derived from simulated fall outcomes and time-to-fall. Accordingly, this study focuses on short-horizon fall-risk estimation from a single static posture.

We operationalize the proposed initial-state formulation by developing a physics-based bed–human simulation framework and using it to create a dataset that pairs sampled initial postures with their subsequent outcomes in simulation. We represent each initial state using skeleton-joint coordinates and train the regression models to predict this continuous risk target from a single static posture. Through these simulation experiments, we clarify the predictive value and limitations of relying solely on static initial posture information for bed-related fall prediction. A practical advantage of this formulation is that, under matched simulator conditions, risk could be estimated from a single observed pose without requiring several seconds of temporal context [22].

The main contributions of this study are summarized as follows. First, we present a physics-based simulation pipeline to systematically generate random initial postures on a bed and label them based on fall-from-bed outcomes. Second, we formulate the initial-state risk assessment as a supervised regression task that learns a continuous target derived from simulated fall outcomes and time-to-fall from a single static posture. Third, we experimentally analyze the feasibility and limitations of this initial-state fall-risk regression formulation, including posture-wise generalization and the effect of dataset construction, without relying on temporal motion sequences.

## 2. Related Works

**Bed-exit/fall detection and in-bed monitoring in real environments.** Many studies on smart wards and elderly-care settings focus on detecting bed-exit events or falls using real sensors such as RGB/depth cameras and mattress/pressure sensors. These methods use temporal sequences and consider the task as event detection or intention recognition (e.g., raising an alarm when a patient is getting out of bed or after a fall occurs). Recent studies have used infrared/depth sensing to analyze or predict bed-related falls, pressure-based approaches to recognize bed-exit intention, and skeleton-based monitoring systems for early warning in bedside scenarios [13,34,35]. Although these systems are directly motivated by clinical safety, they generally require continuous monitoring of motion dynamics and do not isolate the amount of predictive information contained in a single static initial posture. In contrast, our study investigates initial-state risk prediction and quantifies the fall risk inferred before any motion is observed.
**In-bed pose/mesh estimation under occlusion and privacy constraints.** Other related studies aim to recover in-bed body poses (2D keypoints, 3D pose, or body mesh), often under severe occlusion from bedding and with privacy-aware sensing [7,36]. To address these challenges, recent studies leverage multimodal sensing and fusion (e.g., depth/pressure/long-wave infrared), reconstruct missing modalities, or jointly estimate body representations that better reflect contact and occlusion patterns [37,38,39,40]. These studies primarily focus on improving pose/mesh estimation accuracy in bed-centered environments. Our problem is complementary, that is, a pose representation (2D skeleton) is assumed to be available and a downstream safety task is investigated, predicting a future fall-from-bed risk from a single static in-bed pose.
**Physics-based simulation and synthetic data for bed-centered learning.** Real fall-from-bed datasets are rare; collecting such data is risky and ethically constrained. Thus, simulation and synthetic data have been widely used to scale the bed-centered learning tasks. Existing studies have demonstrated that physics-based or synthetic generation can provide large labeled corpora for in-bed understanding, such as pressure-image-based 3D pose/shape estimation, synthetic depth generation for in-bed pose estimation, and sim-to-real frameworks for in-bed mesh recovery [41,42,43]. Recent surveys further highlight the scarcity of diverse real in-bed datasets and the need for robust training strategies under occlusion and modality gaps [36]. Building on this motivation, our study uses a physics-based bed–human simulator not to label pose but to label outcomes (fall/non-fall) and time-to-fall, enabling supervised learning of initial-state fall-risk regression entirely from physics-based simulator rollouts.

## 3. Methods

**Figure 1:**
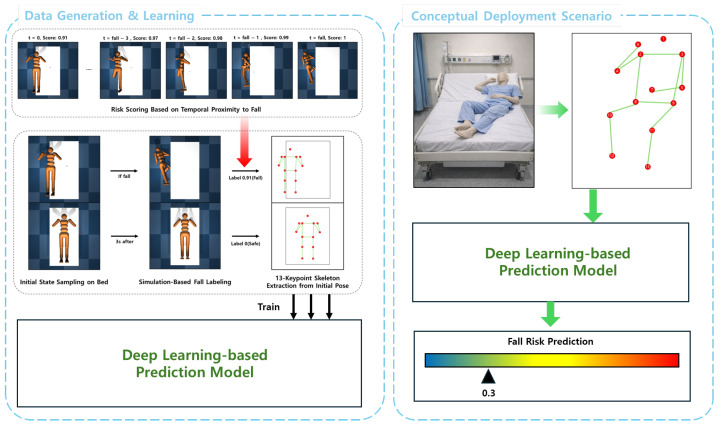
Overall framework of the proposed simulation-based fall risk prediction model from a static skeleton. The right panel illustrates a conceptual deployment scenario without real-world data were collected or evaluated in this study, and all quantitative results are based on physics-based simulation.

We investigate the initial-state fall-from-bed risk prediction using only a single static in-bed pose. As illustrated in Figure 1, the experimental pipeline consists of (i) simulation-based data generation and continuous risk-label construction aa well as (ii) supervised regression model learning that maps a 2D skeleton observation to a continuous fall-risk score. The right panel of Figure 1 illustrates the conceptual deployment of the scenario, where all quantitative evaluations in this experiment are simulated. We let $x\in R^{13\times 2}$ denote a 2D skeleton with 13 keypoints and let $u=\mathrm{vec}\left(x\right)\in R^{26}$ denote its flattened representation used for learning. We use a fixed keypoint set that covers the head and major upper/lower limb landmarks (e.g., shoulder/elbow/wrist and hip/knee/ankle chains), represented as planar coordinates in the bed coordinate frame (in the simulation, keypoints are obtained from predefined body-segment anchor points and used as proxies for anatomical keypoints; see Section 3.1.3). The goal is to predict a continuous risk value $\hat{y}=f_{\theta }\left(u\right)\in R$, where larger values indicate higher risk of a fall event occurring within a fixed rollout time window under uncontrolled dynamics. During the learning process, $f_{\theta }$ is trained using a continuous label y∈[0,1] derived from the time-to-fall in the simulation. During inference, $\hat{y}$ can be optionally thresholded to produce a binary warning signal, but the model is trained as a regression model.

Since real-world fall-from-bed events are rare and difficult to collect safely at a large scale, we generate training data using a physics-based simulator (MuJoCo) by sampling diverse initial poses on a bed, rolling out dynamics, detecting falls, and assigning continuous risk labels based on whether and when a fall occurs. This yields a dataset of paired samples $D={\left\{\left(u_{i},y_{i}\right)\right\}}_{i=1}^{N}$ for supervised learning, where $y_{i}$ is the continuous risk label defined in Equation (4) using the initial-only setting in Equation (6).

Since the predictor observes only a flattened 2D skeleton u rather than the full simulator state, including velocities and contact states, the task is formulated as predicting a continuous fall-risk score from a partial static observation. The target *y* is defined from the subsequent uncontrolled simulator rollout as described in Section 3.1.5.

### 3.1. Simulation-Based Data Generation

#### 3.1.1. Physics-Based Human-in-Bed Simulation

We use MuJoCo (version 3.4.0) to simulate the humanoid body interacting with a rigid bed surface and a floor plane defined in an extensible markup language (XML) model [44]. We use the default MuJoCo humanoid model and represent the bed as a simple box-shaped rigid object, without modeling the mattress compliance or safety rails. Each episode begins with resetting the simulator state and initializing the humanoid above the bed at a fixed drop height. The episode is then rolled out for three seconds with simulator timestep $\Delta t_{\mathrm{sim}}$. We acknowledge that these simplifications might limit the generalizability of the learned risk patterns beyond the current simulator configuration, which is discussed further as a limitation in Section 5.

#### 3.1.2. Initial Pose Sampling and Episode Initialization

For each episode, we sample a target configuration q, coordinates generalized by MuJoCo consisting of (i) a planar location (x,y) on the bed, (ii) a global root orientation, and (iii) joint angles. The planar location is sampled uniformly within a feasible region on the bed while enforcing a margin from the bed edges. We set the root height to a fixed drop height above the bed, which is used only to produce a consistent bed-contact initialization. For global orientation, we employ one of the following strategies:

- **Pose-stratified sampling:** We uniformly select one of four coarse lying posture modes: supine (facing upward), prone (facing downward), left-lateral, and right-lateral. Each mode is implemented as a predefined root-orientation preset. To encourage within-mode diversity and avoid discretized orientations, we add zero-mean Gaussian perturbations to the preset Euler angles (e.g., $\sigma =15^{{}^{\circ}}$ per axis) and convert the perturbed orientation to a quaternion.
- **Fully random sampling:** We sample root Euler angles independently and uniformly from $\left[-180^{{}^{\circ}},180^{{}^{\circ}}\right]$ per axis and convert them to a quaternion.

**Figure 2:**
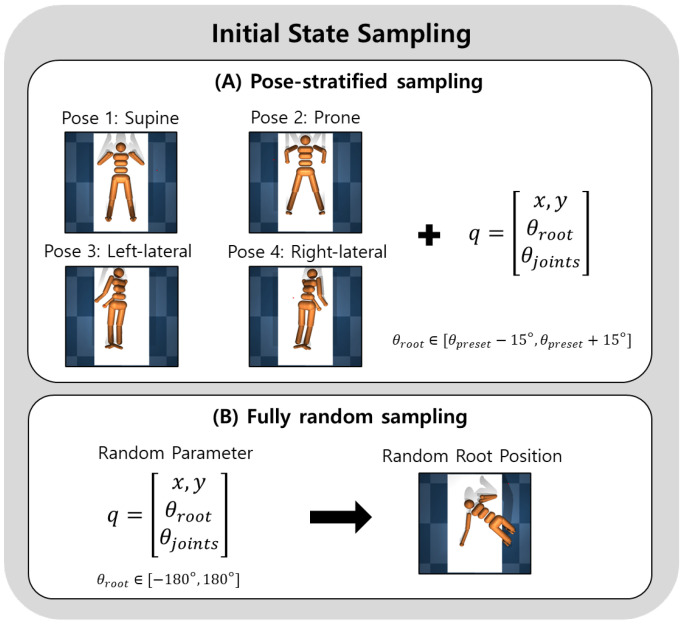
Initial state sampling for diverse in-bed configurations. (**A**) Pose-stratified sampling selects one of four coarse lying postures and perturbs the root orientation. (**B**) Fully random sampling randomizes the planar root position and samples the root and joint angles.

As illustrated in Figure 2, we consider two complementary sampling schemes for constructing diverse initial in-bed configurations. Joint angles are sampled within MuJoCo joint limits when available, and from a default bounded range otherwise, where ball joints are sampled as random unit quaternions. After sampling q, we smoothly place the humanoid in the target configuration by interpolating the simulator state from the reset pose to q over a short pre-pose duration while zeroing velocities at each interpolation step. This produces a controlled initial configuration without residual momentum.

Next, we advance the simulator under gravity until the first contact between the humanoid and the bed is detected or until a timeout is reached. Importantly, we define this first bed-contact instant as the episode start state. At this moment, we freeze the dynamics by setting velocities/accelerations (and controls, if present) to zero and performing a forward pass, and then reset simulation time to t=0 to standardize the rollout origin. All subsequent rollouts used for labeling and learning start from this bed-contact state (i.e., the drop-to-contact phase is treated purely as initialization rather than part of the rollout time window). This drop-to-contact procedure was adopted to avoid geometric interpenetration artifacts that arise when a humanoid is placed directly at a target configuration: lowering the humanoid too far along the vertical axis could cause body segments to clip into the bed geometry, producing physically implausible initial states. By instead dropping the humanoid from above and detecting the first bed-contact instant, we ensure that the episode initial state corresponds to a physically consistent contact configuration. Freezing velocities at that moment further removes residual momentum from the drop phase while preserving the contact-consistent pose. We nonetheless acknowledge that this procedure is inherently artificial and might introduce subtle pose-settling biases at the episode start.

#### 3.1.3. Skeleton Feature Extraction and Normalization

From each simulator state, we extract a 13-keypoint 2D skeleton through mapping the MuJoCo body-segment anchor positions to keypoints. Note that the extracted keypoints are proxy landmarks using segment anchor points for shoulder/hip regions, providing a consistent 2D posture representation in the bed coordinate frame. We let $\left(x_{k},y_{k}\right)$ be the planar coordinates of keypoint k∈{1,…,13}. To obtain a scale- and translation-normalized representation, the coordinates are normalized with respect to the known bed bounds $\left(x_{\min},x_{\max}\right)$ and $\left(y_{\min},y_{\max}\right)$, mapped to [−1,1]:

(1) $$x_{k}^{\prime }\;=\;2\cdot \frac{x_{k}-x_{\min}}{x_{\max}-x_{\min}}-1,\;y_{k}^{\prime }\;=\;2\cdot \frac{y_{k}-y_{\min}}{y_{\max}-y_{\min}}-1.$$

The normalized skeleton is $x=\left[\left(x_{1}^{\prime },y_{1}^{\prime }\right),\ldots ,\left(x_{13}^{\prime },y_{13}^{\prime }\right)\right]\in R^{13\times 2}$, which is flattened to a 26 dimension vector for the training data u,

(2) $$u=\mathrm{vec}\left(x\right)\in R^{26}.$$

Optionally, we compute a per-keypoint validity flag $v_{k}\in \left\{0,1\right\}$ indicating whether the keypoint lies within the bed bounds. These flags can be stored for quality control or ablations; unless it is stated, the regression model uses the coordinates only in Equation (2) (a 2D representation is utilized to facilitate future deployment with practical sensing setups, where estimating reliable 3D joint coordinates in bed environments is often difficult due to occlusion, limited viewpoints, and calibration constraints; in contrast, 2D keypoints could be obtained more robustly from common camera-based pose estimators [45]).

#### 3.1.4. Fall Event Detection

We define a fall event by floor contact within the rollout episode. If any humanoid body part contacts the floor geometry during the rollout, the episode is marked as a fall. To reduce the false positives from incidental contacts, we exclude hand contacts from the fall condition. Episodes that trigger simulator instability warnings are discarded. This exclusion is intended to avoid labeling protective bracing or incidental hand touches near the bed edge as terminal fall events. In the simulator, the hands can contact the floor while the trunk or lower body remains partially supported by the bed, counting such cases as falls would introduce label noise and would overestimate the risk owing to self-stabilizing or partially supported postures [46,47]. Accordingly, hand contact alone is treated as non-terminal unless another body part subsequently contacts the floor within the rollout time window.

#### 3.1.5. Continuous Risk Label from Time-to-Fall

We let $s_{f}$ be the (discrete) simulator step index at which a fall is first detected. The MuJoCo simulation timestep (in seconds) is set as $\Delta t_{\mathrm{sim}}$. To make the discounting behavior independent of the internal simulator frequency and consistent with typical video sampling, we define risk labels on a fixed temporal grid of F=30 frames per second (fps). We convert the simulator step index *s* to a 30 fps frame indexed as follows:

(3) $$n\left(s\right)\;=\;\left\lfloor F\;s\;\Delta t_{\mathrm{sim}}\right\rfloor ,\;n_{f}\;=\;n\left(s_{f}\right),$$

where n(s) is the frame index corresponding to step *s*, and $n_{f}$ is the frame index at the fall time. For a skeleton observed at simulator step *s*, the continuous label is

(4) $$y_{s}\;=\;\left\{\begin{array}{cc} \gamma^{\left(n_{f}-n\left(s\right)\right)}, & \mathrm{if}\;a\;\mathrm{fall}\;\mathrm{occurs}\;\mathrm{at}\;s_{f},\\ 0, & \mathrm{if}\;\mathrm{no}\;\mathrm{fall}\;\mathrm{occurs}\;\mathrm{within}\;\mathrm{the}\;\mathrm{time}\;\mathrm{window}. \end{array}\right.$$

Here, γ∈(0,1) is the per-frame discount factor defined on the 30 fps grid (we use γ=0.99 unless stated otherwise).

This assigns higher risk to observations closer to the fall event on a standardized time scale and provides a graded supervision signal for regression [48]. The target can also be viewed as a bounded time-to-event score:

(5) $$\gamma^{\left(n_{f}-n\right)}\;=\;\exp\left(-\lambda \Delta t\right),\;\lambda =-F\log\gamma ,\;\Delta t=\frac{n_{f}-n}{F}.$$

Thus, the proposed label is a monotone exponential transformation of the remaining time-to-fall. In this study, we always use the initial state (s=0), that is,

(6) $$y\;=\;y_{0}\;=\;\left\{\begin{array}{cc} \gamma^{n_{f}}, & \mathrm{if}\;a\;\mathrm{fall}\;\mathrm{occurs},\\ 0, & \mathrm{otherwise}. \end{array}\right.$$

This directly matches the initial static pose problem setting, and each sampled episode contributes exactly one training pair (u,y).

#### 3.1.6. Class Balancing and Dataset Construction

To mitigate data imbalance, we construct datasets with approximately equal numbers of fall and non-fall episodes by continuing simulation until the target count per class is reached. Each stored sample contains the 26D normalized skeleton coordinates (and optional validity flags) and the corresponding label *y*. Data are stored as CSV files for the subsequent training. Under the initial-only setting adopted in the main experiments, each stored pair (u,y) is later used as one supervised regression sample from a single static partial observation.

### 3.2. Fall Risk Regression Models

Given a partial observation $u\in R^{26}$, the models in this section estimate a continuous fall-risk score from a single static skeleton. All compared models use the same input representation and target construction defined in Section 3.1.5 and differ only in the model architecture.

#### 3.2.1. Multilayer Perceptron (MLP) Regression Model

Our primary predictor is an MLP trained to regress the continuous target defined in Section 3.1.5. The MLP takes the flattened and normalized skeleton vector $u\in R^{26}$ (Equation (2)) as input and outputs a scalar estimate $\hat{y}\in R$ of the fall-risk score. The MLP is implemented as a sequence of fully connected layers with nonlinearities:

(7) $$\hat{y}\;=\;f_{\theta }\left(u\right)\;=\;{\mathrm{MLP}}_{\theta }\left(u\right).$$

The full model configurations and training settings are reported in Table 1 and Table 2.

#### 3.2.2. Additional Regression Models

To compare model families for the fall-risk regression, we additionally evaluate several alternative function approximators (e.g., random forest (RF), convolutional neural network (CNN) models, and graph convolutional network (GCN) and long short-term memory (LSTM) models) using the same skeleton representation and target construction. These models are used as benchmarking baselines for a common supervised regression setting and are described in Section 4.

#### 3.2.3. Regression Loss and Optimization

We train each model to regress the continuous target *y* using the mean squared error (MSE):

(8) $$L\left(\theta \right)\;=\;\frac{1}{N}\sum_{i=1}^{N}{\left(f_{\theta }\left(u_{i}\right)-y_{i}\right)}^{2}.$$

Minimizing this MSE objective fits the model to the continuous risk labels generated from simulator rollouts. The optimization details and training hyperparameters are elaborated in Section 4.

## 4. Experimental Results

### 4.1. Evaluation Scope: Simulation-Only Validation

We evaluate the proposed initial-state fall-from-bed risk prediction entirely within a physics-based simulator. Since fall-from-bed events are rare and ethically difficult to capture at a large scale, simulation provides a controlled environment for generating diverse initial postures with deterministic fall-event labeling (floor contact) and time-to-fall measurements under a fixed physics model. Accordingly, the experiments quantify (i) the predictive information contained in a single static initial 2D skeleton when test-time dynamics match the training simulator and (ii) how the dataset construction and model selection affect the generalization to held-out simulated test sets.

### 4.2. Experimental Protocol

#### 4.2.1. Episode Definition and Simulation Procedure

Each episode was initialized by placing the humanoid at a fixed drop height (2.0 m) above the bed and interpolating to a sampled target configuration for a short pre-pose duration (1.0 s) while setting velocities to zero. We then advanced dynamics under gravity until the first bed contact was detected (or a timeout occurred). We defined this first bed-contact instant as the episode-beginning state by freezing dynamics (setting velocities/accelerations and controls, if present, to zero) and resetting simulation time to t=0. From this standardized initial state, we rolled out uncontrolled dynamics for a fixed time window T=3.0 s to determine fall outcomes and time-to-fall (see Section 3.1.4 and Section 3.1.5).

#### 4.2.2. Labels and Training Samples

We defined the binary fall-event label as c=1 if any body part contacted the floor within the time window, and c=0 otherwise (hand contacts excluded; Section 3.1.4). For the model training, we used the continuous discounted risk label y∈[0,1] derived from time-to-fall on a fixed F=30 fps grid (Equation (4)) and each episode contributed exactly one training sample (u,y) from the initial state episode (t=0 in Equation (6)).

#### 4.2.3. Dataset

Unless stated, the dataset was class-balanced by constructing approximately equal numbers of fall and non-fall episodes. We split the dataset into training and validation subsets (e.g., 9:1). Model selection and threshold calibration were performed on the validation dataset only, without using the test dataset. To evaluate the generalization across the canonical lying orientations, a fixed pose-balanced test set of 50,000 initial samples was constructed through uniformly sampling four coarse posture modes (supine, prone, left-lateral, right-lateral), and both overall and per-posture subgroup metrics were investigated [49]. This pose-balanced test set was designed as a controlled evaluation set for comparing models under consistent orientation coverage rather than for reproducing real-world bedside prevalence. Likewise, pose-stratified sampling was adopted as a coverage-oriented data-generation strategy, since fully random initialization after the drop-to-contact initialization could produce an overly concentrated synthetic posture distribution dominated by straight/supine-like settled poses.

#### 4.2.4. Metrics

Threshold-free metrics—area under the receiver operating characteristic curve (AUROC) and area under the precision-recall curve (AUPRC)—were computed using the binary label *c* with the predicted risk $\hat{y}$ as a ranking score. Thresholded metrics (accuracy, precision, recall, and F1-score) were computed after selecting a threshold τ on the validation set through maximizing F1-score. Regression error, the mean squared error (MSE), was computed against the continuous label *y* for regression-trained models. Note that AUPRC depends on the class prevalence in the evaluation set [50]; we therefore primarily used AUROC to compare discrimination performance across settings and reported AUPRC as an additional prevalence-dependent indicator.

#### 4.2.5. Model Implementation and Training Details

To improve reproducibility, we report the implementation and training details of all compared models here. Unless otherwise stated, the neural models (MLP, CNN, GCN, and LSTM) were trained to regress the continuous target *y* using the MSE loss in Equation (8). The model used for the test evaluation was selected according to the validation criterion used in our experiments. Table 1 summarizes the shared training settings, and Table 2 summarizes the model-specific configurations. RF was implemented using the Scikit-learn (version 1.7.2) random-forest regressor.

### 4.3. Experiment 1: Model Comparison Through Pose-Balanced Testing

We first compared the model families under a fixed data construction protocol to establish a standardized reference performance for subsequent experiments. Unless stated, each model was trained on a class-balanced dataset with N=100,000 initial-only samples constructed with the pose-stratified sampling (see Section 3.1.2) and evaluated on a fixed pose-balanced test set of 50,000 initial samples.

Table 3 shows that the MLP-based predictor achieved the best overall performance among the compared models. Compared with the RF, MLP improves AUROC by +0.0103 and F1 score by +0.0177 while reducing MSE (from 0.0447 to 0.0374), suggesting that the simple non-linear MLP model on the 26D coordinate vector is sufficient to capture the posture-dependent risk information in this experiment. Despite its high performance, GCN does not outperform MLP, indicating that explicit skeleton-topology bias (i.e., redefined joint connectivity encoded as a graph, as in GCN) is not necessarily beneficial [51,52] when inputs are low-dimensional and already normalized to a bed-centric coordinate system. The 1D CNN-based predictor had the worst performance among the neural network models, which is consistent with the fact that the convolutional process over non-kinematic keypoint ordering does not align well with the underlying kinematic structure [53,54].

To assess the additional predictive value of temporal context over a single static pose, we included an LSTM baseline trained on short sequences of T=5 consecutive frames from the episode start. The LSTM achieved an AUROC of 0.9750±0.0008 and F1-score of 0.9133±0.0017, which are comparable with the MLP (0.9755±0.0006 and 0.9138±0.0015, respectively). Notably, the LSTM has access to richer temporal information than the single-frame MLP, yet it achieves comparable performance. This suggests that, under the current simulator dynamics and evaluation setting, temporal context beyond a single static frame did not yield additional predictive benefit, supporting the feasibility of the single-frame approach as a computationally efficient alternative.

To ensure that the overall gains were not driven by a single canonical orientation, we analyzed the per-posture results for the best-performing model, MLP, in Table 4. The performance was consistently high across all four posture modes (AUROC between 0.9706 and 0.9822). The supine posture mode achieved the highest AUROC and lowest MSE, suggesting that the risk patterns might be more stable to be trained under this orientation in the current simulation setup. The left-lateral posture mode was slightly more challenging than the others (lowest AUROC and highest MSE of the four), which might be attributed to the increased variability in the edge proximity and body support patterns under the lateral configurations. Overall, the relatively consistent performance across postures could indicate that the trained prediction model generalizes across the various canonical lying orientations rather than overfitting to a single posture mode.

### 4.4. Experiment 2: Ablation on Sampling Strategy (Random vs. Pose-Stratified)

We isolated the effect of the initial-state coverage through comparing the fully random sampling with the pose-stratified sampling with four posture presets and perturbations while keeping the total number of training samples (N=100,000) and using the same training schedule and model (MLP).

As shown in Table 5, pose-stratified sampling improves overall performance: AUROC increases from 0.9668 to 0.9755 and MSE decreases from 0.0454 to 0.0374. A notable pattern is the precision–recall trade-off under a validation-selected threshold: random sampling attained slightly higher precision (0.9295) but substantially lower recall (0.8614), leading to a worse F1-score. This suggests that fully random initialization may under-cover certain risky configurations in the pose-balanced evaluation set, causing the model to behave more conservatively (fewer positives) when tuned for F1-score. In contrast, pose-stratified sampling explicitly enforces coverage across canonical orientations, reducing distribution mismatch and improving recall and overall F1-score [55].

### 4.5. Experiment 3: Training-Set Size Scaling

**Figure 3:**
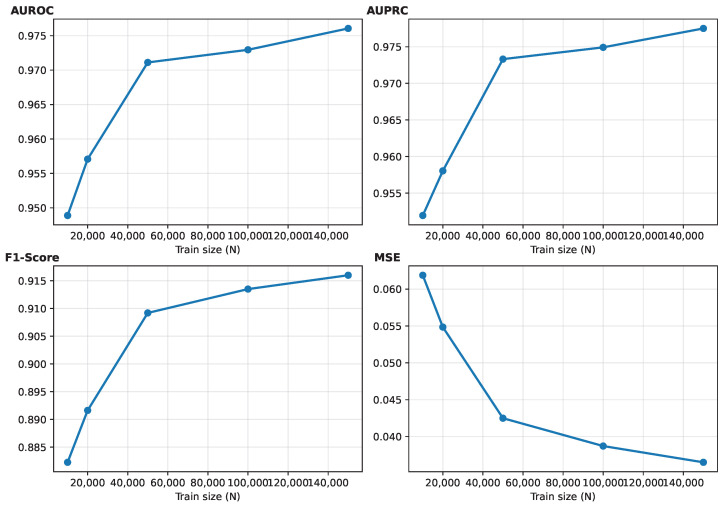
AUROC, AUPRC, F1-score, and MSE performances corresponding to the training-set size, N∈{10,000, 20,000, 50,000, 100,000, 150,000} on the pose-balanced test set.

We analyzed data efficiency when training MLP on datasets of different sizes N∈{10,000, 20,000, 50,000, 100,000, 150,000} using the same protocol as Experiment 1. Figure 3 summarizes AUROC, AUPRC, F1-score, and MSE corresponding to the training set size. As expected, AUROC and AUPRC improved with the size of the training dataset, and MSE reduced, confirming that the broad coverage of the simulated initial states with more training data benefits the model generalization on a held-out pose-balanced test set. The curve also illustrates that AUROC and AUPRC stabilized earlier than MSE. This pattern suggests that ranking-oriented and calibration-sensitive metrics may improve at different rates as training data increase. These results provide a practical guideline on the number of simulated initial states needed to reach targeted performance levels under fixed simulators.

### 4.6. Experiment 4: Preliminary Cross-Domain Evaluation on Real Fall Data

To assess whether to the real-world data, we applied the trained MLP predictor directly to a subset of the FallVision dataset [56] without retraining. FallVision is a publicly available RGB video dataset comprising fall and non-fall videos recorded from the voluntary participants using handheld devices. From the bed-fall subset, we manually selected the 100 videos for which bed-edge configurations were clearly identifiable. For each selected video, a single frame was randomly sampled from the pre-fall period and manually annotated with 13 keypoints following the same skeleton definition used in our simulator. As summarized in Table 6, the resulting evaluation set was class-balanced with 50 fall and 50 non-fall samples. The predictor was evaluated using the threshold calibrated on the simulated validation set. All metrics reported in Table 7 are averaged five random seeds, and AUROC is as the primary discriminative metric the calibrated threshold not be optimal for the real-data distribution.

The model achieved an AUROC of 0.8175±0.0188 on the real-data evaluation set compared to 0.9755±0.0006 on the matched simulated test set. The performance decrease confirms the presence of a Sim-to-Real gap, attributable to differences in the body morphology, bed geometry, and the distribution shift between the simulator episode-start poses and real pre-fall postures. Nonetheless, an AUROC above 0.80 suggests that the partially capture geometrically discriminative patterns present in the real pre-fall postures, motivating future work on domain adaptation and score recalibration.

## 5. Discussion

This study investigated fall-from-bed risk prediction from a single static in-bed posture using a physics-based bed–human simulation framework. By generating large-scale labeled episodes with deterministic fall detection (floor contact) and time-to-fall-measurements, we evaluated the learnability of initial-state-based risk prediction under controlled settings.

**Key findings from simulation-only validation.** Among various models, we observed strong predictive performance using only a 13-keypoint 2D skeleton extracted at a standardized episode initial state.

The MLP-based predictor achieved the best results on the pose-balanced test set, indicating that substantial fall-related information is contained in static posture under matched simulator dynamics. Per-posture evaluation further showed consistently high performance across supine, prone, and lateral posture configurations, suggesting that the trained prediction model is not biased to a single canonical orientation.

**What information is likely extracted from a static skeleton?** Even without temporal context, a bed-centric skeleton implicitly encodes geometric cues that affect subsequent uncontrolled dynamics, such as the proximity of major body segments to bed edges, asymmetry between left/right lateral configurations, and overall body orientation that might influence sliding or rolling movements. The strong performance of a simple MLP on the 26D coordinate vector suggests that, in this simulation, these cues could be estimated via relatively low-dimensional nonlinear decision boundaries, while additional structural inductive bias (e.g., GCN topology) offers limited incremental benefit (see Table 3). This also helps explain why a 1D-CNN baseline underperformed; that is, the convolution over an arbitrary keypoint ordering does not naturally encode the kinematic structure.
**Impact of dataset construction and coverage.** The sampling ablation highlights that the coverage of canonical lying orientations matters for the model generalization. Pose-stratified sampling improves AUROC (0.9668→0.9755) and reduces MSE (0.0454→0.0374) compared to fully random initialization (see Table 5), consistent with the reduced distribution mismatch against a pose-balanced evaluation set. We therefore interpret pose-stratified sampling primarily as a coverage-oriented simulation design choice rather than as an estimate of real-world posture prevalence. In our simulator setup, fully random initialization followed by the drop-to-contact initialization can yield an overly concentrated synthetic distribution dominated by straight/supine-like settled poses while under-covering prone and lateral configurations. The pose-stratified scheme was adopted to avoid such concentration and to ensure representation across common coarse lying orientations. Accordingly, the strongest results in Table 3 should be interpreted as performance under a matched, pose-controlled simulator distribution rather than as evidence of robustness to broader distribution shifts.
**Labeling choice and interpretation of regression error.** We labeled the level of fall-risk using a discounted time-to-fall label on a 30 fps grid in Equation (4). With γ=0.99 and T=3 s (i.e., at most 90 frames), fall episodes obtained labels in approximately $\left[\gamma^{90},1\right]\approx \left[0.41,1\right]$ while non-fall episodes were labeled as 0. Therefore, the regression partially differentiates the no-fall events from the fall-within-3 s events with a bounded continuous scale for fall cases, which could yield high AUROC/AUPRC even when fine-grained calibration is imperfect. For deployment, additional calibration analysis (e.g., reliability curves, expected calibration error, and operating-point selection under application-specific costs) would be required to translate $\hat{y}$ into actionable alarms.
**Metric caveats under real-world prevalence.** Our training and test sets were class-balanced to ensure learnability and fair comparison of the various models. In addition, the main quantitative results were obtained with pose-stratified training and a pose-balanced simulated test set. Although these choices were useful for isolating whether a single static posture contains predictive information under controlled simulator conditions, they do not establish robustness to shifts in class prevalence, posture frequency, patient morphology, bed configuration, or sensing quality. In practice, real fall-from-bed events are rare, and the distribution of in-bed postures is unlikely to be balanced across canonical orientations. Accordingly, the reported AUROC/AUPRC and thresholded metrics should be interpreted as performance under a matched evaluation distribution, and deployment performance might degrade when the posture mix or event prevalence differs from the simulated training conditions.
**Limitations of the simulator and sensing assumptions.** Several limitations to the suggested model should be acknowledged. First, the trained prediction model was designed with specific simulator assumptions, that is, a rigid bed without mattress compliance or rails, default humanoid model, and fixed contact/friction parameters. These simplifications might have shaped the reported risk patterns in ways specific to the current simulator configuration, and the transferability of the learned predictor to different physical setups remains to be verified. Second, skeletons were extracted without sensing noise; in real bedside environments, 2D keypoints may be missing or jittery due to the occlusion by blankets, limited viewpoints, and pose-estimation errors [7,57]. Third, dynamics were uncontrolled and time window-limited; real patients actively move and bed environments vary depending on facilities. Accordingly, our study should be interpreted as an upper-bound feasibility study under matched simulation dynamics rather than direct clinical validity [58]. Fourth, the drop-to-contact initialization procedure, while adopted to prevent geometric interpenetration artifacts, involves an artificial velocity-freezing step at first bed contact; the potential influence of this procedure on the learned risk patterns remains to be empirically characterized. Fifth, the best performing setting combines pose-stratified training with a pose-balanced test set, which is appropriate for controlled feasibility analysis but leaves robustness to broader covariate shifts unresolved. Sixth, the 2D skeleton representation discards height information, which might be relevant for edge-hanging limb configurations. We adopted 2D keypoints because reliable 3D joint coordinates are difficult to obtain in bed environments due to occlusion, limited viewpoints, and the absence of depth sensing in common camera-based setups. A systematic comparison with 3D skeleton input therefore requires additional sensing infrastructure and is left for future work.
**Future work toward sensor-based smart healthcare application.** To better align with the real sensing environment, future work will incorporate (i) domain randomization over bed geometry and physical parameters [59,60,61], (ii) robustness training with the skeleton keypoint noise and missing patterns, (iii) score calibration using real datasets with appropriate ethical oversight, and (iv) further real-data validation with larger and more diverse datasets to strengthen the Sim-to-Real gap analysis initiated in Experiment 4. These steps would enable a more reliable evaluation of the initial-state risk scoring as a component of AI-enabled bedside monitoring systems.

## 6. Conclusions

This study investigated the feasibility of fall-from-bed risk prediction from a single static initial posture within a controlled physics-based simulation framework. The task was formulated as supervised regression under uncontrolled dynamics, using a continuous risk label derived from simulated fall outcomes and time-to-fall. Using a 13-keypoint 2D skeleton representation normalized to a bed-centric coordinate frame, we trained and evaluated multiple regression models for this task. On a fixed pose-balanced test set, the MLP-based predictor achieved the best overall performance. We further demonstrated that pose-stratified sampling improves model generalization compared with fully random initialization, and increasing the number of simulated training samples consistently improves test performance. Although the results demonstrate that a static posture contains meaningful predictive signals under controlled simulator dynamics, the study is limited by simplified physics assumptions and absence of real sensing noise and active patient motion. A preliminary cross-domain evaluation on a publicly available real-fall dataset further confirmed the presence of a Sim-to-Real gap, underscoring the need for domain adaptation before practical deployment. These findings should therefore be interpreted as evidence of learnability under controlled and partially matched simulator distributions, rather than as a demonstration of robustness to real-world distribution shifts. Future work will focus on the sim-to-real transfer through physics domain randomization, robustness to noisy/missing skeleton keypoints, and calibration under realistic class prevalence, followed by real-world verification under appropriate ethical and safety protocols.

## Figures and Tables

**Table 1:** Shared training settings for the neural models (MLP, CNN, GCN and LSTM).

Item	Setting
Loss	Mean squared error (Equation (8))
Optimizer	Adam
Initial learning rate	$1\times 10^{-3}$
Validation split ratio	0.1
Batch size	512
Maximum epochs	50
Random seed(s)	{41, 42, 43, 44, 45}

**Table 2:** Model-specific configurations.

Model	Configuration
MLP	**Input:** 26D flattened skeleton vector.
	**Architecture:** 26 → 256 → 128 → 1.
	**Activation:** ReLU after hidden layers.
	**Dropout:** p=0.1.
CNN	**Input:** reshaped to 2×13 tensor.
	**Encoder:** Conv1d(2,32,3) → ReLU → Conv1d(32,64,3) → ReLU → AvgPool.
	**Head:** 64 → 64 → 1 (ReLU).
	**Dropout:** p=0.1.
GCN	**Input:** 13 nodes, 2D features.
	**Graph:** fixed skeleton graph with self-loops, normalized adjacency.
	**Encoder:** 2 layers (hidden dim 64) with ReLU + dropout.
	**Head:** 832 → 256 → 128 → 64 → 1.
	**Dropout:** p=0.1.
RF	**Library:** RandomForestRegressor (scikit-learn).
	**Parameters:** n_estimators = 300, max_depth = None, random_state = seed (varied per run).
	**Others:** default values.
LSTM	**Input:** sequence of T=5 frames, reshaped to (5,26) per sample. Frames are sampled at 30 fps from the episode start (t=0 to t=4)
	**Encoder:** 1-layer LSTM (hidden dim 64).
	**Head:** 64 → 1 (last hidden state).
	**Dropout:** p=0.1.

**Table 3:** Benchmark tests on the pose-balanced simulation test set (50,000).

Model	AUROC	AUPRC	Accuracy	Precision	Recall	F1-Score	MSE
RF	0.9652±0.0002	0.9680±0.0001	0.8953±0.0013	0.8895±0.0078	0.9030±0.0075	0.8961±0.0005	0.0447±0.0001
MLP	0.9755±0.0006	0.9771±0.0005	0.9137±0.0013	0.9129±0.0059	0.9149±0.0073	0.9138±0.0015	0.0374±0.0003
CNN	0.9579±0.0013	0.9613±0.0010	0.8824±0.0032	0.8749±0.0125	0.8929±0.0105	0.8837±0.0020	0.0508±0.0009
GCN	0.9692±0.0007	0.9713±0.0008	0.9022±0.0017	0.8968±0.0054	0.9092±0.0059	0.9029±0.0017	0.0420±0.0008
LSTM	0.9750±0.0008	0.9767±0.0007	0.9134±0.0015	0.9141±0.0031	0.9126±0.0052	0.9133±0.0017	0.0367±0.0005

**Table 4:** Per-posture subgroup performance using the best-performing model (MLP) on the pose-balanced simulated test set (50,000).

Posture	AUROC	AUPRC	Accuracy	Precision	Recall	F1-Score	MSE
supine	0.9822±0.0001	0.9783±0.0003	0.9287±0.0006	0.9103±0.0059	0.9207±0.0071	0.9154±0.0008	0.0339±0.0003
prone	0.9706±0.0005	0.9744±0.0005	0.9049±0.0008	0.9147±0.0097	0.9020±0.0124	0.9082±0.0017	0.0393±0.0004
left_lateral	0.9713±0.0013	0.9737±0.0013	0.9049±0.0019	0.9092±0.0045	0.9010±0.0064	0.9051±0.0021	0.0389±0.0006
right_lateral	0.9765±0.0010	0.9818±0.0007	0.9161±0.0028	0.9164±0.0092	0.9349±0.0077	0.9255±0.0021	0.0375±0.0005

**Table 5:** Ablation: random vs. pose-stratified sampling (using MLP with 100,000 training data points).

Sampling	AUROC	AUPRC	Accuracy	Precision	Recall	F1-Score	MSE
Random	0.9668±0.0004	0.9699±0.0003	0.8979±0.0018	0.9295±0.0068	0.8614±0.0105	0.8941±0.0028	0.0454±0.0010
Pose-stratified	0.9755±0.0006	0.9771±0.0005	0.9137±0.0013	0.9129±0.0059	0.9149±0.0073	0.9138±0.0015	0.0374±0.0003

**Table 6:** Summary of the real-data evaluation subset extracted from the FallVision dataset [56].

Label	Fall	Non-Fall	Total
Samples	50	50	100

**Table 7:** Preliminary cross-domain evaluation results on the FallVision dataset (MLP, no retraining, N=100).

Model	AUROC	AUPRC	Accuracy	Precision	Recall	F1-Score
MLP	0.8175±0.0188	0.8677±0.0087	0.7380±0.0311	0.7190±0.0355	0.7840±0.0261	0.7497±0.0265

## Data Availability

The raw data supporting the conclusions of this article will be made available by the authors on request.

## References

[1] Anderson O., Boshier P., Hanna G. (2011). Interventions designed to prevent healthcare bed-related injuries in patients. Cochrane Database of Systematic Reviews.

[2] Morse J.M., Gervais P., Pooler C., Merryweather A., Doig A.K., Bloswick D. (2015). The safety of hospital beds: Ingress, egress, and in-bed mobility. Glob. Qual. Nurs. Res..

[3] Islam M., Nayan N.M., Islam A., Sikder S., Rashel M.R., Alam M.Z. (2024). Recent advancements of computer vision in healthcare: A systematic review. IEIE Trans. Smart Process. Comput..

[4] Bowers B., Lloyd J., Lee W., Powell-Cope G., Baptiste A. (2008). Biomechanical evaluation of injury severity associated with patient falls from bed. Rehabil. Nurs. J..

[5] Asghari M., Elali K., Toosizadeh N. (2025). The effect of age on ankle versus hip proprioceptive contribution in balance recovery: Application of vibratory stimulation for altering proprioceptive performance. Biomed. Eng. Lett..

[6] Ocagli H., Lanera C., Borghini C., Khan N.M., Casamento A., Gregori D. (2024). In-bed monitoring: A systematic review of the evaluation of in-bed movements through bed sensors. Informatics.

[7] Liu S., Yin Y., Ostadabbas S. (2019). In-bed pose estimation: Deep learning with shallow dataset. IEEE J. Transl. Eng. Health Med..

[8] Jähne-Raden N., Kulau U., Marschollek M., Wolf K.H. (2019). INBED: A highly specialized system for bed-exit-detection and fall prevention on a geriatric ward. Sensors.

[9] Fernández-Bermejo Ruiz J., Dorado Chaparro J., Santofimia Romero M.J., Villanueva Molina F.J., del Toro García X., Bolaños Peño C., Llumiguano Solano H., Colantonio S., Flórez-Revuelta F., López J.C. (2022). Bedtime monitoring for fall detection and prevention in older adults. Int. J. Environ. Res. Public Health.

[10] Park D., So K., Prabhakar S.K., Kim C., Lee J.J., Sohn J.H., Kim J.H., Lee S.H., Won D.O. (2025). Early warning score and feasible complementary approach using artificial intelligence-based bio-signal monitoring system: A review. Biomed. Eng. Lett..

[11] Zhao F., Cao Z., Xiao Y., Mao J., Yuan J. (2019). Real-time detection of fall from bed using a single depth camera. IEEE Trans. Autom. Sci. Eng..

[12] Shim J., Shim M.H., Baek Y.S., Han T.D. (2011). The development of a detection system for seniors’ accidental fall from bed using cameras. Proceedings of the 5th International Conference on Ubiquitous Information Management and Communication.

[13] Meng F., Liu T., Meng C., Zhang J., Zhang Y., Guo S. (2024). Method of bed exit intention based on the internal pressure features in array air spring mattress. Sci. Rep..

[14] Bai D., Ho M.C., Mathunjwa B.M., Hsu Y.L. (2023). Deriving multiple-layer information from a motion-sensing mattress for precision care. Sensors.

[15] Bauer P., Kramer J.B., Rush B., Sabalka L. (2017). Modeling bed exit likelihood in a camera-based automated video monitoring application. Proceedings of the 2017 IEEE International Conference on Electro Information Technology (EIT).

[16] Kwolek B., Kepski M. (2014). Human fall detection on embedded platform using depth maps and wireless accelerometer. Comput. Methods Programs Biomed..

[17] Zhou H., Zhu W. (2024). Vision-based Multi-task Hybrid Model for Teacher-Student Behavior Recognition in Classroom Environment. IEIE Trans. Smart Process. Comput..

[18] Broadley R.W., Klenk J., Thies S.B., Kenney L.P., Granat M.H. (2018). Methods for the real-world evaluation of fall detection technology: A scoping review. Sensors.

[19] Klenk J., Schwickert L., Palmerini L., Mellone S., Bourke A., Ihlen E.A., Kerse N., Hauer K., Pijnappels M., Synofzik M. (2016). The FARSEEING real-world fall repository: A large-scale collaborative database to collect and share sensor signals from real-world falls. Eur. Rev. Aging Phys. Act..

[20] Casilari E., Silva C.A. (2022). An analytical comparison of datasets of Real-World and simulated falls intended for the evaluation of wearable fall alerting systems. Measurement.

[21] Moon Y.B., Oh T.H. (2024). Label-efficient learning methods for computer vision applications. IEIE Trans. Smart Process. Comput..

[22] Wang Z., Armin M.A., Denman S., Petersson L., Ahmedt-Aristizabal D. (2021). Video-based inpatient fall risk assessment: A case study. Proceedings of the 2021 43rd Annual International Conference of the IEEE Engineering in Medicine & Biology Society (EMBC).

[23] Woltsche R., Mullan L., Wynter K., Rasmussen B. (2022). Preventing patient falls overnight using video monitoring: A clinical evaluation. Int. J. Environ. Res. Public Health.

[24] Woolrych R., Zecevic A., Sixsmith A., Sims-Gould J., Feldman F., Chaudhury H., Symes B., Robinovitch S.N. (2015). Using video capture to investigate the causes of falls in long-term care. Gerontologist.

[25] Schulz B.W., Lee W.E., Lloyd J.D. (2008). Estimation, simulation, and experimentation of a fall from bed. J. Rehabil. Res. Dev..

[26] Thompson A.K., Bertocci G.E. (2013). Paediatric bed fall computer simulation model development and validation. Comput. Methods Biomech. Biomed. Eng..

[27] Pascoletti G., Catelani D., Conti P., Cianetti F., Zanetti E.M. (2019). Multibody models for the analysis of a fall from height: Accident, suicide, or murder?. Front. Bioeng. Biotechnol..

[28] Thompson A., Bertocci G. (2014). Pediatric bed fall computer simulation model: Parametric sensitivity analysis. Med. Eng. Phys..

[29] Yoder A.J., Petrella A.J., Farrokhi S. (2021). Sensitivity of a subject-specific ankle sprain simulation to extrinsic versus intrinsic biomechanical factors. Front. Bioeng. Biotechnol..

[30] Santos V.J., Bustamante C.D., Valero-Cuevas F.J. (2009). Improving the fitness of high-dimensional biomechanical models via data-driven stochastic exploration. IEEE Trans. Biomed. Eng..

[31] Igual R., Medrano C., Plaza I. (2013). Challenges, issues and trends in fall detection systems. Biomed. Eng. Online.

[32] Gutiérrez J., Rodríguez V., Martin S. (2021). Comprehensive review of vision-based fall detection systems. Sensors.

[33] Mobsite S., Alaoui N., Boulmalf M., Ghogho M. (2023). Semantic segmentation-based system for fall detection and post-fall posture classification. Eng. Appl. Artif. Intell..

[34] Chen L.B., Chang W.J., Yang T.C. (2025). BedEye: A Bed Exit and Bedside Fall Warning System Based on Skeleton Recognition Technology for Elderly Patients. IEEE Access.

[35] Ishizu F., Tajima T., Abe T. (2023). Analysis and Prediction of Patient Falls from Beds Using an Infrared Depth Sensor. Sens. Mater..

[36] Yazici Z.A., Colantonio S., Ekenel H.K. (2024). In-bed pose estimation: A review. Proceedings of the 2024 IEEE International Conference on Pervasive Computing and Communications Workshops and Other Affiliated Events (PerCom Workshops).

[37] Tandon A., Goyal A., Clever H.M., Erickson Z. (2024). Bodymap-jointly predicting body mesh and 3d applied pressure map for people in bed. Proceedings of the IEEE/CVF Conference on Computer Vision and Pattern Recognition.

[38] Zhu Y., Xiao M., Xie Y., Xiao Z., Jin G., Shuai L. (2024). In-bed human pose estimation using multi-source information fusion for health monitoring in real-world scenarios. Inf. Fusion.

[39] Dayarathna T., Muthukumarana T., Rathnayaka Y., Denman S., De Silva C., Pemasiri A., Ahmedt-Aristizabal D. (2023). Privacy-preserving in-bed pose monitoring: A fusion and reconstruction study. Expert Syst. Appl..

[40] Nahin S.K., Acharjee S., Saha S., Das A., Hossain S., Haque M.A. (2024). Human sleeping pose estimation from IR images for in-bed patient monitoring using image processing and deep learning techniques. Heliyon.

[41] Clever H.M., Erickson Z., Kapusta A., Turk G., Liu K., Kemp C.C. (2020). Bodies at rest: 3D human pose and shape estimation from a pressure image using synthetic data. Proceedings of the IEEE/CVF Conference on Computer Vision and Pattern Recognition.

[42] Ochi S., Miura J. (2022). Depth-based in-bed human pose estimation with synthetic dataset generation and deep keypoint estimation. Proceedings of the European Conference on Computer Vision.

[43] Gao J., Zheng C., Jeni L.A., Erickson Z. (2025). DiSRT-In-Bed: Diffusion-based sim-to-real transfer framework for in-bed human mesh recovery. Proceedings of the IEEE/CVF Conference on Computer Vision and Pattern Recognition.

[44] Todorov E., Erez T., Tassa Y. (2012). Mujoco: A physics engine for model-based control. Proceedings of the 2012 IEEE/RSJ International Conference on Intelligent Robots and Systems.

[45] Cao Z., Simon T., Wei S.E., Sheikh Y. (2017). Realtime multi-person 2D pose estimation using part affinity fields. Proceedings of the IEEE Conference on Computer Vision and Pattern Recognition.

[46] Lamb S.E., Jørstad-Stein E.C., Hauer K., Becker C., Prevention of Falls Network Europe and Outcomes Consensus Group (2005). Development of a common outcome data set for fall injury prevention trials: The Prevention of Falls Network Europe consensus. J. Am. Geriatr. Soc..

[47] Maki B.E., McIlroy W.E. (1997). The role of limb movements in maintaining upright stance: The “change-in-support” strategy. Phys. Ther..

[48] Yèche H., Pace A., Ratsch G., Kuznetsova R. (2023). Temporal label smoothing for early event prediction. Proceedings of the International Conference on Machine Learning.

[49] Koh P.W., Sagawa S., Marklund H., Xie S.M., Zhang M., Balsubramani A., Hu W., Yasunaga M., Phillips R.L., Gao I. (2021). Wilds: A benchmark of in-the-wild distribution shifts. Proceedings of the International Conference on Machine Learning.

[50] Saito T., Rehmsmeier M. (2015). The precision-recall plot is more informative than the ROC plot when evaluating binary classifiers on imbalanced datasets. PLoS ONE.

[51] Shi L., Zhang Y., Cheng J., Lu H. (2019). Two-stream adaptive graph convolutional networks for skeleton-based action recognition. Proceedings of the IEEE/CVF Conference on Computer Vision and Pattern Recognition.

[52] Chen Z., Li S., Yang B., Li Q., Liu H. (2021). Multi-scale spatial temporal graph convolutional network for skeleton-based action recognition. Proceedings of the AAAI Conference on Artificial Intelligence.

[53] Caetano C., Brémond F., Schwartz W.R. (2019). Skeleton image representation for 3d action recognition based on tree structure and reference joints. Proceedings of the 2019 32nd SIBGRAPI Conference on Graphics, Patterns and Images (SIBGRAPI).

[54] Yang Z., Li Y., Yang J., Luo J. (2018). Action recognition with spatio–temporal visual attention on skeleton image sequences. IEEE Trans. Circuits Syst. Video Technol..

[55] Shimodaira H. (2000). Improving predictive inference under covariate shift by weighting the log-likelihood function. J. Stat. Plan. Inference.

[56] Rahman N.N., Mahi A.B.S., Mistry D., Al Masud S.M.R., Saha A.K., Rahman R., Islam M.R. (2025). FallVision: A benchmark video dataset for fall detection. Data Brief.

[57] Karácsony T., Carmona J., Cunha J.P.S. (2025). Blanketgen2-fit3d: Synthetic blanket augmentation towards improving real-world in-bed blanket occluded human pose estimation. arXiv.

[58] Collins G.S., Reitsma J.B., Altman D.G., Moons K.G. (2015). Transparent reporting of a multivariable prediction model for individual prognosis or diagnosis (TRIPOD): The TRIPOD statement. Br. J. Surg..

[59] Tobin J., Fong R., Ray A., Schneider J., Zaremba W., Abbeel P. (2017). Domain randomization for transferring deep neural networks from simulation to the real world. Proceedings of the 2017 IEEE/RSJ International Conference on Intelligent Robots and Systems (IROS).

[60] Peng X.B., Andrychowicz M., Zaremba W., Abbeel P. (2018). Sim-to-real transfer of robotic control with dynamics randomization. Proceedings of the 2018 IEEE International Conference on Robotics and Automation (ICRA).

[61] Chebotar Y., Handa A., Makoviychuk V., Macklin M., Issac J., Ratliff N., Fox D. (2019). Closing the sim-to-real loop: Adapting simulation randomization with real world experience. Proceedings of the 2019 International Conference on Robotics and Automation (ICRA).

